# Supplementary material for: The discovery of novel noncoding RNAs in 50 bacterial genomes
Source: Nucleic Acids Res. 2024 Apr 22;52(9):5152–65. doi: 10.1093/nar/gkae248 (PMC11109978; doi:10.1093/nar/gkae248)
Supplement: gkae248_Supplemental_Files [file gkae248_supplemental_files.zip › Narunsky 50 Genomes RNA Motifs Files.zip!/Publication/53-A_muciniphila/Figures/LRC-53-3-NC_010655_1_IGR_59_1635772_1635926.sto.pdf]

LRC-53-3-NC_010655_1_IGR_59_1635772_1635926.sto.r2r

5′-RA●AURC●GYYCAU●YCR○AAACAA●RR●GGCCACCUGY●●●ARCAGGUGACC●YCAAUAACGGGCRURAGCCCAAYYAAY●●RA○YR●A●●AY●ACUA●YAYAA●●●AYR●●R●●●CGCAAG○○○○○○○○○○○○
